# Image segmentation of plexiform neurofibromas from a deep neural network using multiple b-value diffusion data

**DOI:** 10.1038/s41598-020-74920-1

**Published:** 2020-10-20

**Authors:** Chang Y. Ho, John M. Kindler, Scott Persohn, Stephen F. Kralik, Kent A. Robertson, Paul R. Territo

**Affiliations:** 1 Department of Radiology and Imaging Sciences, Indiana University School of Medicine, Indianapolis, IN USA; 2 Department of Radiology, Texas Children’s Hospital, Houston, TX USA; 3 Department of Pediatrics, Indiana University School of Medicine, Indianapolis, IN USA; 4 Department of Medicine, Indiana University School of Medicine, Indianapolis, IN USA; 5 MRI Department, Indiana University School of Medicine, 705 Riley Hospital Drive, Indianapolis, IN 46202 USA

**Keywords:** Diagnostic markers, Computational biology and bioinformatics, Peripheral neuropathies, Cancer, Cancer imaging

## Abstract

We assessed the accuracy of semi-automated tumor volume maps of plexiform neurofibroma (PN) generated by a deep neural network, compared to manual segmentation using diffusion weighted imaging (DWI) data. NF1 Patients were recruited from a phase II clinical trial for the treatment of PN. Multiple b-value DWI was imaged over the largest PN. All DWI datasets were registered and intensity normalized prior to segmentation with a multi-spectral neural network classifier (MSNN). Manual volumes of PN were performed on 3D-T2 images registered to diffusion images and compared to MSNN volumes with the Sørensen-Dice coefficient. Intravoxel incoherent motion (IVIM) parameters were calculated from resulting volumes. 35 MRI scans were included from 14 subjects. Sørensen-Dice coefficient between the semi-automated and manual segmentation was 0.77 ± 0.016. Perfusion fraction (f) was significantly higher for tumor versus normal tissue (0.47 ± 0.42 vs. 0.30 ± 0.22, *p* = 0.02), similarly, true diffusion (D) was significantly higher for PN tumor versus normal (0.0018 ± 0.0003 vs. 0.0012 ± 0.0002, *p* < 0.0001). By contrast, the pseudodiffusion coefficient (D*) was significantly lower for PN tumor versus normal (0.024 ± 0.01 vs. 0.031 ± 0.005, *p* < 0.0001). Volumes generated by a neural network from multiple diffusion data on PNs demonstrated good correlation with manual volumes. IVIM analysis of multiple b-value diffusion data demonstrates significant differences between PN and normal tissue.

## Introduction

Neurofibromatosis type 1 (NF1) is a common genetic neurocutaneous disease, affecting 1 in 3000 newborn infants^1^. There are over 1000 identified mutations of the *NF1* gene which produces a tumor suppressor protein called neurofibromin^2^. A prototypical NF1 tumor is the plexiform neurofibroma (PN), a neurofibroma variant with tumor cells that spread along multiple nerve fascicles, resulting in an extensive mass of thickened nerve bundles in a proteinaceous matrix. These PNs affect 25–50% of NF1 patients and can occur anywhere there are nerve fibers leading to significant morbidity and mortality depending on the size and location adjacent to vital structures^3^. Approximately 10% of PNs can transform to become malignant peripheral nerve sheath tumors over the lifetime of the patient^3^. Currently, surgical resection is the only available therapy, but is infrequently curative due to extensive invasion, making complete resection difficult^3^. No effective medical therapy currently exists; however, clinical trials are underway to evaluate chemotherapy which shrink tumor or limit growth.

Therefore, accurate measurements of tumor burden are necessary to assess for tumor response. Two dimensional measurements are limited in assessing treatment change because of the common irregularity and extensiveness of PNs. Considerable intra- and interobserver variation has also been demonstrated in two- or three-dimensional manual measurements^4^. Furthermore, in cases with large, extensive PNs throughout the entire body, user fatigue can result in measurement errors^5^. Ideally, automated segmentation of PNs from normal tissue with limited user interaction would decrease intra- and interobserver variability as well as user fatigue. The purpose of this project is to develop a neural network deep learning algorithm to perform semi-automated volume segmentation of PNs based on multiple b-value diffusion weighted MRI and to assess the accuracy of the tumor volume maps by comparing to manual segmentation. Post-segmentation, intravoxel incoherent motion (IVIM) parameters generated from the multiple b-value modeling of data are also compared between PN and background normal tissue.

## Methods

The Indiana University Institutional Review Board approved this prospective study. All NF1 subjects participated with informed consent in a phase II clinical trial of imatinib mesylate (Imatinib) for the treatment of PNs^6^. Inclusion criteria were NF1 patients with at least one plexiform neurofibroma greater than 1 cm in a single dimension, an MRI scan with multiple b-value diffusion weighted imaging (DWI) at baseline, and at least one follow-up MRI at 6-month intervals. All MRI sequences were performed on a 1.5 T scanner (Magnetom Avanto, Siemens Healthineers, Erlangen, Germany) and included 3D T2-weighted spectral attenuated inversion recovery (SPAIR) sequences in the coronal plane (TR = 2200, TE = 200, 1 mm slice thickness, 0 mm gap, FA = 120°, matrix = 448 × 450) as well as diffusion weighted sequences in the axial or sagittal plane with multiple b-values (b = 0, 50, 150, 200, 400, 600, TR = 2137, TE = 68.4, ST = 5 mm, gap = 6.5 mm, FA = 90°) focused on the largest PN. Sagittal or axial orientation was chosen on diffusion weighted sequences for maximum in-plane coverage of the largest PN. For the purposes of comparison to diffusion weighted datasets, modified SPAIR images were reformatted in the same plane as the diffusion dataset at matching 5 mm slice thickness and truncated to match the diffusion anatomical coverage.

### Neural network machine learning

**Figure 1:**
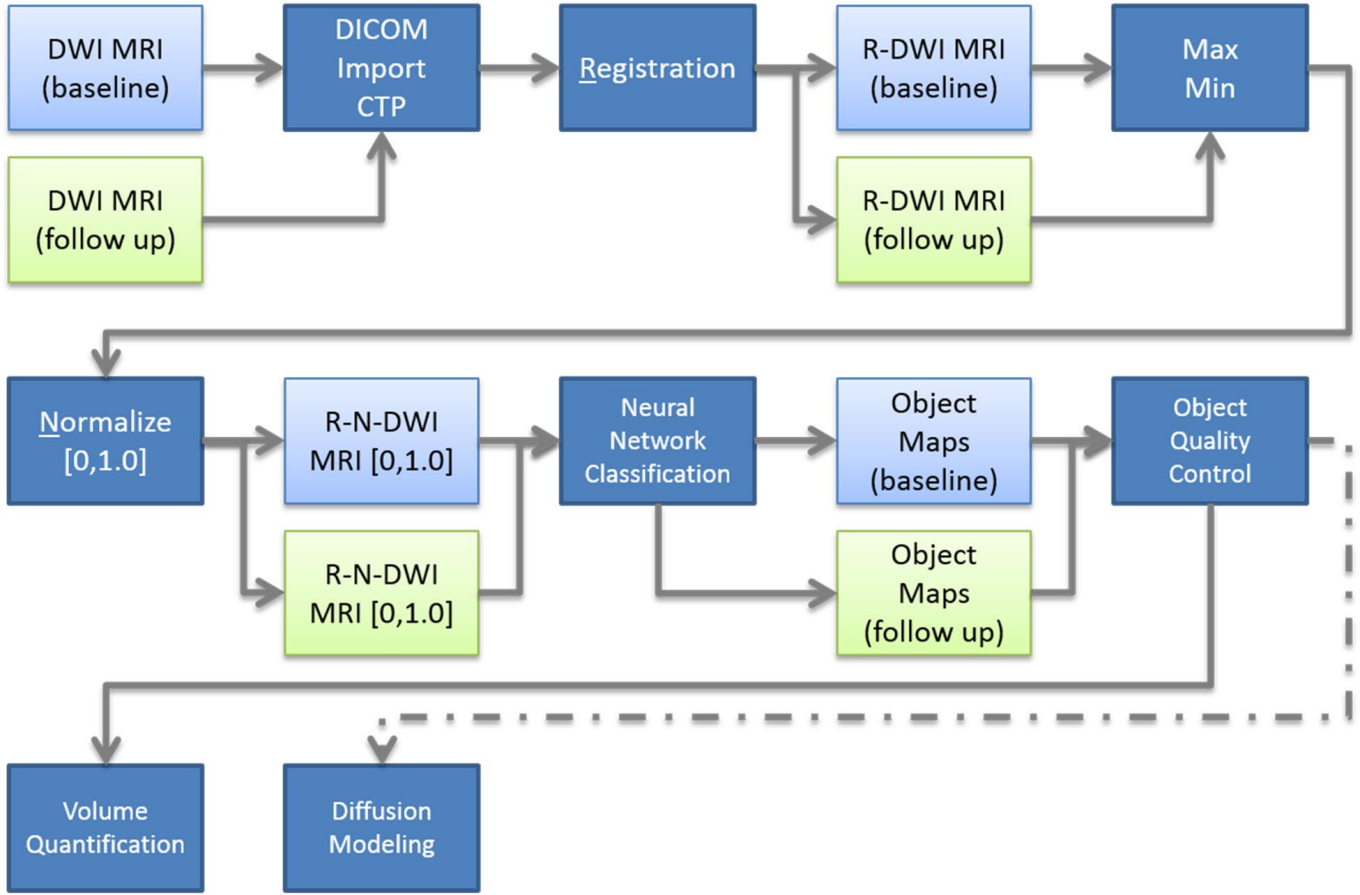
Image processing workflow for Multi-Spectral Neural Network classification. DWI MRI image series from baseline and follow up scans are imported into the Clinical Trials Processor for de-identification and then registered using the normalized entropy mutual information method described previously^7^. Post registration, maximum and minimum intensities were computed, images intensity normalized over the interval [0.0, 1.0], and images were segmented using the MSNN method. Object maps were then individually inspected for classification errors, cleaned (if required), and then submitted to volumetric and IVIM diffusion modeling.

**Figure 2:**
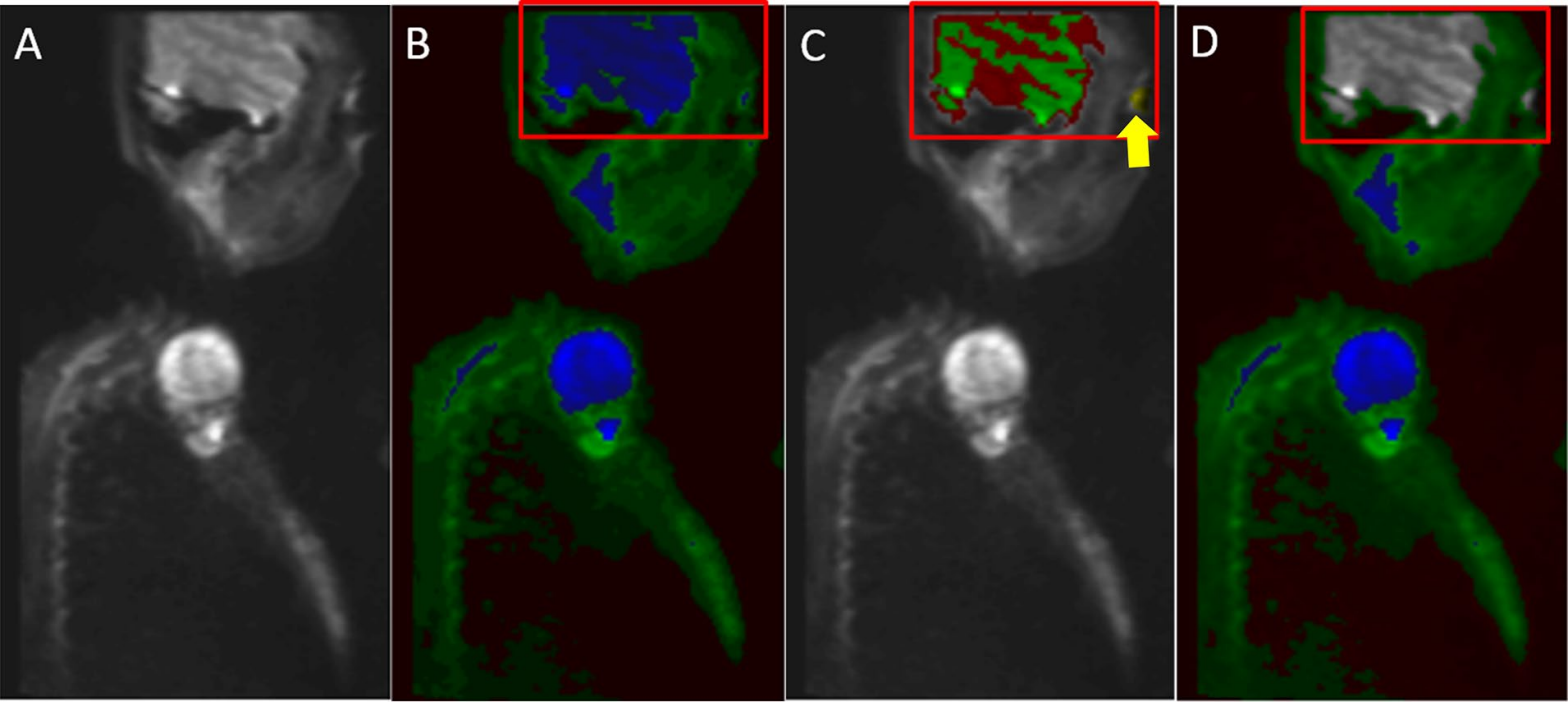
Representative normalized sagittal images of cervical DWI MRI: (**A**) b = 0 DWI image (unclassified); (**B**) Initial Classified Object Map based on the MSNN model described in Fig. 1; normal tissue (green) and tumor (blue); (**C**) Misclassified structures such as Brain (red) and Orbits (yellow arrow) which require exclusion from tumor volume; and (**D**) Quality control corrected normal (green) and tumor (blue).

All diffusion datasets were registered to the baseline time point for each subject using the normalized entropy mutual information method described previously^7^. Images were intensity normalized over the interval [0.0, 1.0] by dividing each image volume by the maximum value of the B0 image set (Fig. 1). Using the multispectral neural network (MSNN) modeling tool available in Analyze 12.0 (AnalyzeDirect, Overland Park, KS)^8^, model priors for each “class” were constructed by defining regions of interest for normal (i.e. non-tumor) tissue, NF1 lesions, and “background” (i.e. remainder) that were extracted across all registered normalized multi-b value (0, 50, 100, 150, 200, 400, 600) DWI images. The selection of hidden layers was based on an empirical analysis of the model, where the minimum probability was fixed at 0.1, alpha probability was set to 0.25, and epochs set at 200 according to the guidance from the manufacturer. Under these conditions, the number of hidden layers was varied systematically from 3 to 20 layers and the degree of segmentation assessed for over classification. Once the number of layers were selected, the number of epochs were systematically varied from 100 to 1000, keeping the number of hidden layers, minimum probability, and alpha probability constant. Once optimized, the multi-b value voxel series for each class were then applied to a feed-forward neural network consisting of 15 hidden layers each having 3 nodes (Appendix Fig. 8), for training purposes, with 200 epochs. In addition, the recommended minimum probability of 0.1 and an alpha probability of 0.25 were used for voxel classification. The minimum probability represents the likelihood that any given voxel belongs to a defined class; voxels with values less than this probability are left unclassified. Similarly, the alpha probability is a confidence-weighting parameter which controls the strength of the relaxation during iterative optimization. Due to design limitations of the MSNN tool, only a single training set could be defined as part of the model priors; therefore, we randomly selected an NF1 subject from the population to serve as the training set for all subsequent segmentation, and was excluded from the remaining analysis. Post-training, classification of the remaining normalized multi-b value images for each subject were then performed generating 3D object maps for each region in the image series. Each resulting volume map identified as tumor by the neural network was then reviewed and manually edited for obvious erroneous anatomy by a trained imaging scientist (SP) which commonly included bladder, brain, eyes, and spinal fluid (Fig. 2). These misclassified anatomical structures were excluded from the tumor volume.

### Manual segmentation analysis

As a comparison to manual segmentation, a neuroradiology fellow (JK) manually traced all plexiform neurofibromas on the registered SPAIR images in consensus with the senior neuroradiologist (CH). Measurements were made on a commercially available image analysis software (MIM Encore 6.9.3, MIM Software INC, Cleveland, OH, https://www.mimsoftware.com/nuclear_medicine/encore) using a freehand ROI tool. The volumes of the manual measurements were summated for each MRI exam and used for analysis. Bland–Altman plots were performed between the MSNN volume results and corresponding manual segmentation results. Linear regression and Sørensen–Dice coefficient was used to analyze correlation and concordance between manual tumor volumes and neural network results.

### IVIM analysis

Neural network generated volume maps were also used to generate average b-value series for IVIM analysis according to the bi-exponential model proposed by Le Bihan et al*.*^9^:

1 $$\frac{S\left( b \right)}{{S\left( 0 \right)}} = f \times e^{{ - bD^{*} }} + \left( {1 - f} \right) \times e^{ - bD}$$

where *S*, *b*, *f*, D^*^, and *D* are the raw signal, b-value, perfusion fraction, pseudo-diffusion (mixed diffusion) coefficient (sec/mm^2^), and true molecular diffusion (slow diffusion) coefficient (sec/mm^2^), respectively. Calculation of IVIM parameters were performed on a molecular kinetic modeling software (*eLucidate*, Paul R. Territo, Ph.D, Indiana University, Indianapolis, USA), where the uniformly weighted sum-of squares difference (SSD) between the objective function (Eq. 1) and normalized b-value series was optimized via Simulated Annealing (relative SSD, tolerance 1e−15). IVIM parameters were compared across available scanning time points at baseline, 6 months and 12 months, between PN tumors and normal structures excluding the bladder, brain, eyes and spinal fluid. Significant differences of IVIM parameters were assessed between all PN tumor and normal tissue using a two-tailed paired T-test, while a One-way ANOVA was used to assess for significant changes in IVIM parameters for PNs between baseline, 6-month and 12-month scans. In all cases, significance was taken at the *p* ≤ 0.05 level.

### Ethical approval

All procedures performed in studies involving human participants were in accordance with the ethical standards of the institutional and/or national research committee and with the 1964 Helsinki declaration and its later amendments or comparable ethical standards.

### Informed consent

Written informed consent was obtained from all individual participants included in the study.

## Results

A total of 35 MRI scans were included from 14 subjects (mean age 22.6 years; range 2–51 years; 11 females, 3 males). Location for the largest PN included: head and neck (9 subjects), chest (3 subjects), abdomen and pelvis (1 subject), and right thigh (1 subject).

**Figure 3:**
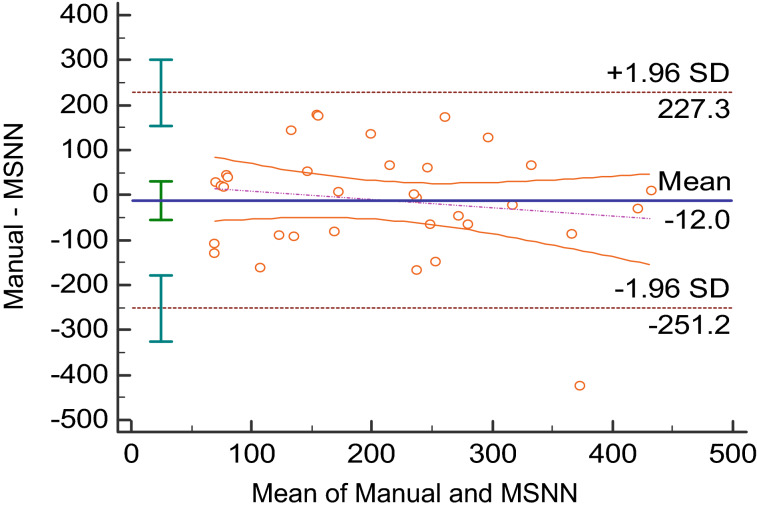
Bland–Altman analysis of MSNN and manual segmentation volumes in milliliters. The purple dot and dash line represents the linear regression of the differences with 95% confidence interval lines.

**Figure 4:**
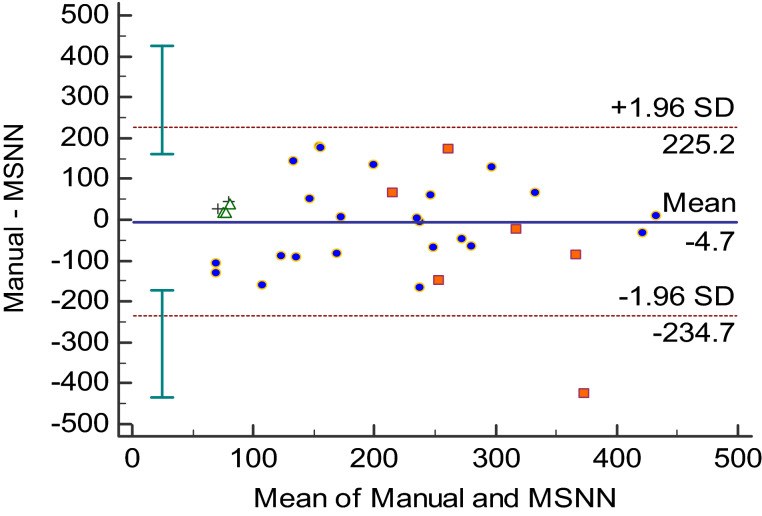
Bland–Altman analysis between MSNN and manual segmentation volumes in milliliters. Each data point represents an anatomic region for the measured PNs. Cross = abdomen/ pelvis, triangle = thigh, circle = head/neck and square = chest.

**Figure 5:**
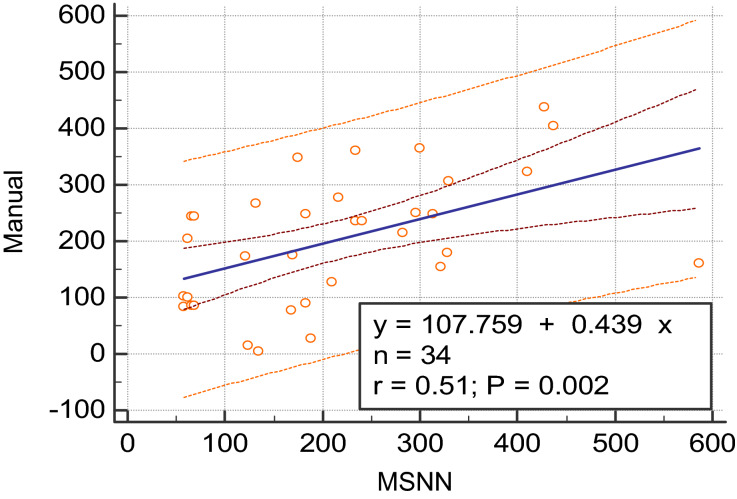
Linear regression analysis of manual versus MSNN volumes with trend line and 95% confidence intervals.

On average, a neural network case including manual definition of tumor classes with removal of obvious normal anatomy took 6 min, while manually measuring the PNs took an average of 18 min. Bland–Altman plots of the MSNN versus manual segmentation results are also shown in Figs. 3 and 4. Raw values for the Bland Altman plots are given in Table 1. Linear regression (Fig. 5) showed significant correlation (r = 0.51, *p* = 0.002). The Sørensen–Dice coefficient was 0.77 ± 0.016 (± SEM, range 0.54–0.96).

**Table 1:** Individual subject volumes in cubic millimeters for the manual and MSNN segmentation methods.

MRI scan (#)	Manual (mm^3^)	MSNN (mm^3^)	Average (mm^3^)	Difference (mm^3^)	% Difference	Region
1	84,116	57,239	70,678	26,877	38	Abd/Pelvis
2	102,200	57,267	79,734	44,933	56	Abd/Pelvis
3	172,495	120,543	146,519	51,952	35	Head/Neck
4	175,431	169,303	172,367	6127	4	Head/Neck
5	100,013	62,270	81,142	37,743	47	Thigh
6	85,172	65,845	75,508	19,327	26	Thigh
7	85,172	68,423	76,798	16,748	22	Thigh
8	204,618	62,270	133,444	142,348	107	Head/Neck
9	242,986	65,845	154,416	177,141	115	Head/Neck
10	242,827	68,423	155,625	174,403	112	Head/Neck
11	365,620	299,650	332,635	65,970	20	Head/Neck
12	405,175	437,408	421,292	− 32,232	− 8	Head/Neck
13	437,522	427,553	432,538	9969	2	Head/Neck
14	305,228	328,925	317,077	− 23,697	− 7	Chest
15	323,213	410,551	366,882	− 87,339	− 24	Chest
16	77,969	168,348	123,159	− 90,379	− 73	Head/Neck
17	89,170	182,879	136,024	− 93,709	− 69	Head/Neck
18	27,533	188,696	108,114	− 161,162	− 149	Head/Neck
19	276,930	216,959	246,944	59,971	24	Head/Neck
20	234,409	240,454	237,432	− 6045	− 3	Head/Neck
21	236,166	234,250	235,208	1916	1	Head/Neck
22	178,562	328,011	253,287	− 149,449	− 59	Chest
23	160,167	586,325	373,246	− 426,159	− 114	Chest
24	266,998	132,493	199,746	134,504	67	Head/Neck
25	360,918	234,274	297,596	126,643	43	Head/Neck
26	347,271	175,144	261,207	172,127	66	Chest
27	247,957	183,138	215,547	64,820	30	chest
28	215,193	282,491	248,842	− 67,298	− 27	Head/Neck
29	247,391	314,026	280,709	− 66,635	− 24	Head/Neck
30	249,562	296,156	272,859	− 46,594	− 17	Head/Neck
31	127,238	210,280	168,759	− 83,043	− 49	Head/Neck
32	154,225	322,093	238,159	− 167,868	− 70	Head/Neck
33	84,116	57,239	70,678	26,877	38	Abd/Pelvis
34	4138	133,978	69,058	− 129,840	− 188	Head/Neck
35	14,717	123,338	69,027	− 108,621	− 157	Head/Neck

In addition, the average, difference and percent difference between the segmentation methods used in the Bland–Altman analysis are provided as well for comparison.

**Figure 6:**
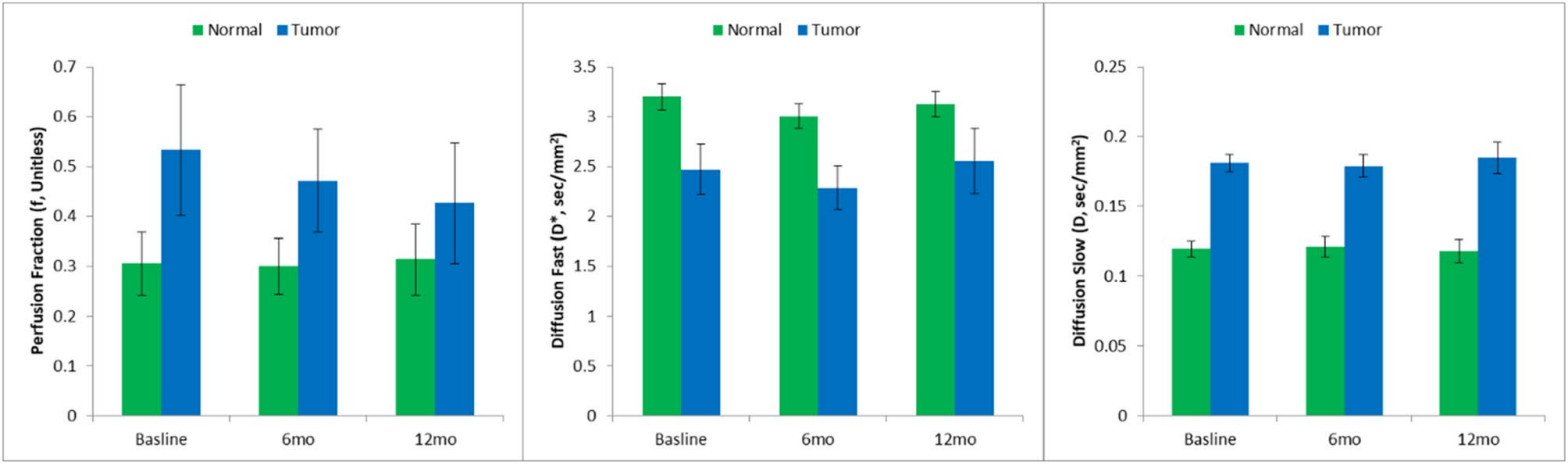
Results for IVIM Kinetic Modeling: Perfusion Fraction (f, unitless); Diffusion Fast (D*, s/mm^2^); Diffusion Slow (D, s/mm^2^) for baseline, 6 and 12 months post Imatinib treatment in normal tissue and NF1 tumors.

All IVIM modeling showed a very high degree of fit across the three time points studied for normal (non-tumor; R = 0.999 ± 0.0001, 0.999 ± 0.0002, 0.998 ± 0.0002) and PN tumors (R^2^ = 0.999 ± 0.0001, 0.999 ± 0.0004, 0.998 ± 0.0005). Moreover, there was a significant difference for all IVIM parameters between normal tissue and PN tumors. Perfusion fraction (i.e. f) was significantly higher for tumor versus normal tissue (0.47 ± 0.42 vs. 0.30 ± 0.22, *p* = 0.02), similarly, D was significantly higher for PN tumor versus normal tissue (0.0018 ± 0.0003 vs. 0.0012 ± 0.0002, *p* < 0.0001). By contrast, D* was significantly lower for PN tumor versus normal tissue (0.024 ± 0.01 vs. 0.031 ± 0.005, *p* < 0.0001). Despite the differences in tissue IVIM parameters, there was no significant change from Imatinib treatment baseline (pre-treatment) when comparing tissues through time (Fig. 6).

## Discussion

**Figure 7:**
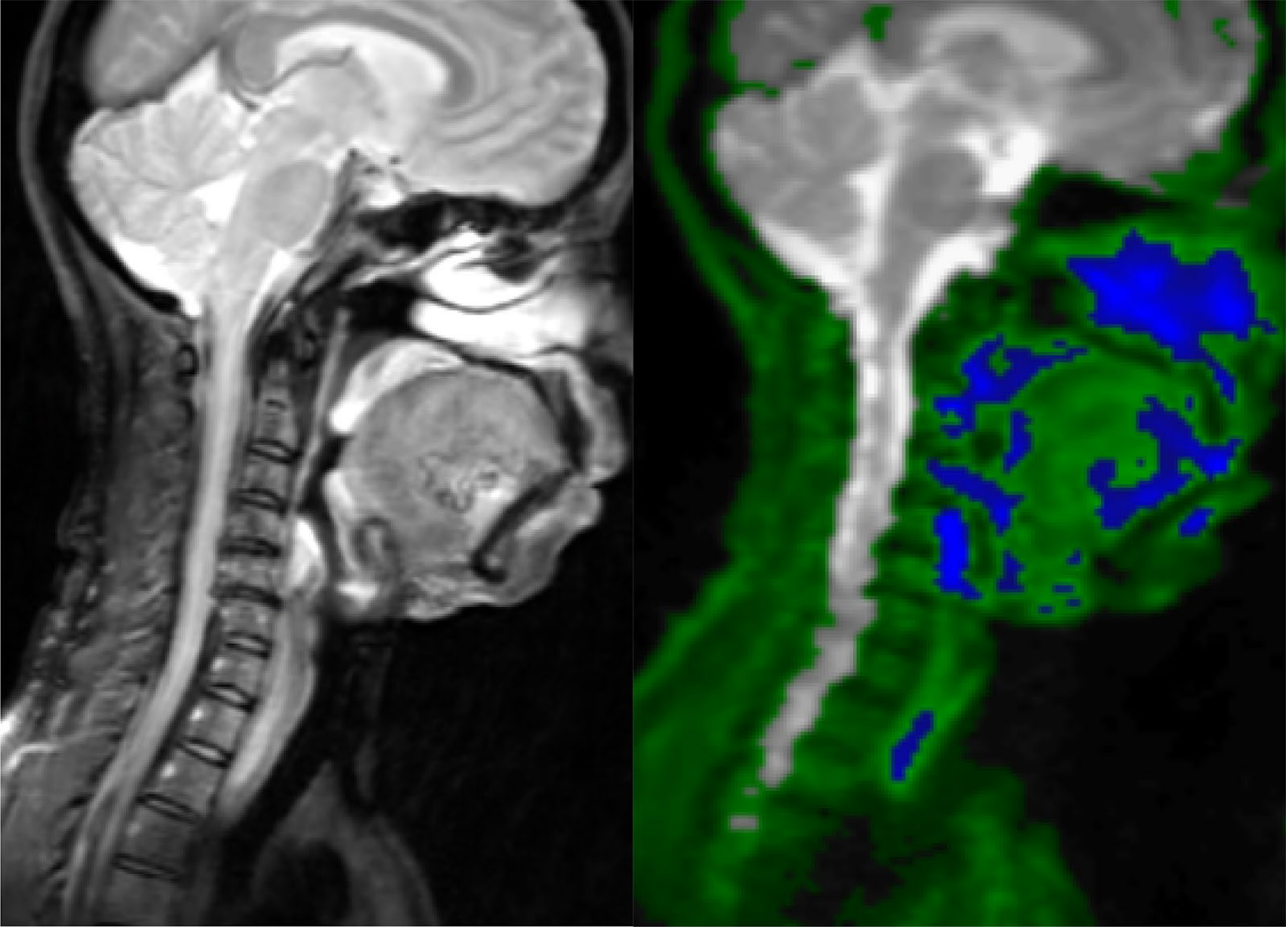
51-year-old female with NF1. (**A**) Sagittal SPAIR image of the head and neck. (**B**) Sagittal neural network map shows erroneous identification of nasal mucosa, palatine tonsils and airway as tumor tissue (blue).

We evaluated the accuracy of semi-automated tumor volumes generated by a deep neural network based on diffusion weighted imaging with multiple b-values. The semi-automated volumes had a 77% concordance rate from Sørensen–Dice analysis compared to manually measured volumes. The Bland Altman plot by scan anatomy showed that discordance was greatest in several of the head and neck and chest cases. This is likely caused by PNs which were in close proximity with the lungs, skull base, and paranasal sinuses, locations highly prone to susceptibility artifact on echo planar imaging (Fig. 7). Although we were able to achieve a good concordance rate with Sørensen–Dice analysis, the inclusion of these areas likely prevented even higher concordance. Similarly, spinal and paraspinal tumors may also be affected by artifact on echo planar imaging, however, several neck and abdomen/pelvis exams had paraspinal location tumors which had better concordance.

Previous semi-automated segmentation of PNs focused on signal intensity from STIR images with either a signal intensity or histogram-based algorithm to identify tumor borders with or without additional spatial connectivity algorithms. Poussaint et al.^10^ used a commercially available image analysis software which requires a user to click on the center of a lesion and then drag until the ROI encompasses the whole lesion. The software is able to compute the edge based on best-guess of the lesion border where signal intensity values are expected to change rapidly; however, in cases where the intensity gradient is low, the edge detection will fail resulting in inaccurate tumor volume^10^. Moreover, since this approach is slice based, it requires repeated application of the seed value for every slice. Finally, the user must then edit the borders with a separate tool. Combined, this approach is both labor intensive and subject to human error, and in has limited use in a clinical setting. Solomon et al.^11^ used a histogram matching method that requires initial user ROI placement on a tumor for each slice, which again is labor intensive. To overcome these limitations, Cai et al.^12^ used a dynamic threshold level set technique which requires users to identify each lesion which then automatically expand in three-dimensional space based on lesion versus background histograms. This approach, which overcomes the slice limitations of previous investigators, still requires that a trained expert identify each tumor in the image volume. Finally, Weizman et al.^13^ also uses a STIR histogram matching algorithm which requires a one-time training requiring manual volumetric analysis of a tumor and identifying of a small frame including most of the tumor and a large frame which includes tumor and background. In the segmentation phase, the user is required to scribble over the tumor, which can then propagate in 3D space; however, like all the described methods, manual error correction of STIR hyper-intense normal structures adjacent to tumor is required.

Comparatively, our method using a deep learning neural network does require a single training phase but afterwards does not require user input in identifying tumors. However, manual user correction of normal anatomy is required, particularly for head and neck cases as brain, eyes and spinal fluid were consistently erroneously classified as tumor despite the training phase (Fig. 2). Diffusion characteristics for these normal structures were too similar to PNs to be properly classified as normal background anatomy. The time necessary for a trained imaging scientist to process the images and delete normal anatomy took on average 6 min compared to an average of 18 min for manual volumetric analysis. Furthermore, Weizman et al. determined that for complex cases, up to 35 min per case would be required for manual segmentation^13^. If one expands this to the typical case load encountered by radiologists at our institution, this amounts to 0.007–0.014 FTE, or $3500–7000/case in annualized cost savings.

Previous works on this topic measured inter-rater volume concordance and repeatability for method assessment. We were not able to perform repeatability because of the lack of user input in the creation of tumor volumes. Once trained, the neural network will generate identical volume maps for the same case, leading to perfect repeatability and concordance for the same case. We did not assess inter-rater manual concordance as measuring large PN volumes in our datasets would likely lead to greater discordance from user fatigue^5,14^. Ultimately no true gold standard exists unless manual volume measurements were performed by a trained expert resistant to fatigue. In clinical trials, change in the volume of tumor burden may be more helpful than accurate tumor volumes, provided consistent inclusion of false positive volumes. Although we did have follow-up MRI scans, these were not performed with the same exact tumor coverage or at the same time and had 6-month intervals with ongoing chemotherapy which would limit a test for method precision for different scans on the same subject.

An ideal gold standard in PN segmentation for clinical trials would require accuracy and precision in addition to a minimal amount of user input to lessen user fatigue. With advances in processor technology and big data, computer deep learning has been applied to various categories of medical imaging including anatomy identification and segmentation^15^, as well as computer aided detection and diagnosis^16^. Our study is the first and only to our knowledge to apply deep learning for semi-automated volume segmentation for PNs in the NF1 population using multiple b-value DWI data. Further investigation in limiting the false positive volumes identified may be accomplished using spin echo-based diffusion techniques without susceptibility distortion. Other quantitative data may also be helpful such as signal intensity, histogram analysis, perfusion or a combined multi-parametric approach for deep learning as have been used for other oncology applications^17^.

Our study is also the first to our knowledge in the literature to assess for IVIM parameters on PN tumors. We were not able to detect treatment effects with imatinib mesylate in IVIM parameters of PN across the timepoints of baseline, 6 month and 12 months after treatment, which suggests that this chemotherapeutic agent may not influence tissue diffusion at the time points studied. Again, a comparison with volumetric analysis using the neural network data was not possible due to lack of consistent inclusion of anatomy on diffusion sequence acquisition. However, we did detect significant differences for all IVIM parameters compared with normal (non-tumor) tissue after removal of brain, bladder, eyes, and spinal fluid from the object maps. Normal background tissue was primarily connective tissue, muscle, bone, solid organs and bowel. Interestingly, PN tumor had significantly higher f, but lower D* compared to normal tissues. The f correlates with blood volume and the D* theoretically correlates with blood flow^18^, suggesting that PNs have a higher total blood volume but slower overall blood flow^19^. These data are consistent with pathological reports of biopsy samples taken from PN subjects when stained for smooth muscle actin, with diffuse plexiform neurofibromas demonstrating the highest vessel density compared to other subtypes of neurofibromas^20^. Although the qualitative detection of PNs compared to background normal tissue is not usually difficult for a trained expert on STIR images, the ability of IVIM to quantitatively differentiate PNs from background tissue may be an alternative approach to aid the neuroradiologist in determining optimal treatment planning and response to therapy.

Finally, the current approach has some limitations. The MSNN model implementation is fixed, limiting its extension to more advanced modeling approaches. For instance, convolutional neural networks have been developed and used successfully for image segmentation^21^. In addition, the current established tool also fixes the training model to a single set of images (i.e. one multi-b value DWI set), preventing additional models and thus limits accuracy. Since the current tool limits the number of model training sets to a single series, one approach may be to perform iterative random selection of the subject to serve as the training set, and then repeat the analysis to evaluate biases that result from the model (i.e. “jack-knifing”). In addition, the MSNN tool does not permit selection of alternative activation functions (i.e. sigmoid, ELU, ReLU, leaky ReLU, tanh, etc.), or the ability to control the level of connections and/or recursions across layers, which limits the broad application of this tool for complex imaging studies. Despite these limitations, the current tool was chosen based on its broad availability, computational efficiency, ease of use for establishing training and analysis sets, and ability to translate to clinical research operations. Based on the initial promising results, these studies can then serve as the basis for custom software tools that overcome the aforementioned limitations. These factors, along with the reasonable high accuracy, suggest that this approach has merit and warrants additional investigation as a time saving tool in clinical practice.

## Conclusion

IVIM parameters can significantly differentiate PNs from normal background tissue. Deep learning of multiple b-value DWI data from a neural network successfully generated volume maps in a semi-automated process. When compared with manual measurements, the semiautomated volumes had a Sørensen–Dice coefficient of 77%. However, PNs in anatomy typically affected by susceptibility artifact within the head, neck and chest limit greater concordance and further work is needed to develop this technology into a routine clinical tool.

## Figures and Tables

**Figure 8:**
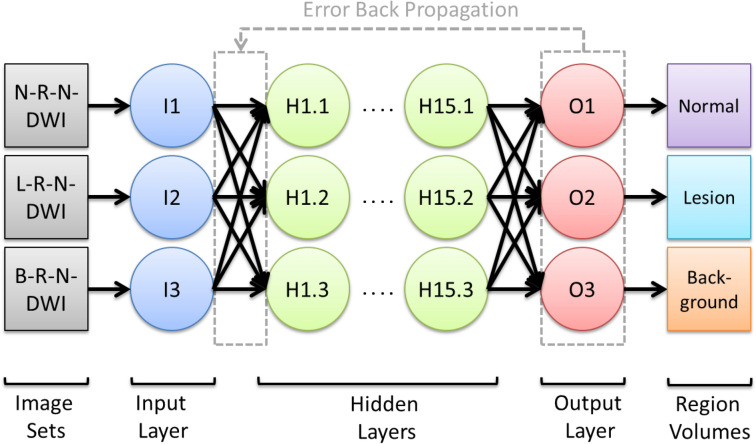
Multi-Spectral Neural Network (MSNN) Classifier Diagram. Normalized Diffusion Weighted Images (DWI) b-Value (0, 50, 100, 150, 200, 400, 600) serve as inputs to the model, where nodal weights are applied to the hidden and output layers to yield output voxels which are classified as normal, tumor, or background once the model is trained. In all cases, training sets represented a random NF1 subject from the population which has been manually segmented by a trained expert. In the input layer N-, L-, and B-prefixes represent the Normal, Lesion, and Background inputs, respectively, while R-N in each set refers to Registered-Normalized images.

## Appendix

See Fig. 8.

## Abbreviations

PN: Plexiform neurofibroma
NF1: Neurofibromatosis type 1
MSNN: Multispectral neural network
IVIM: Intravoxel incoherent motion
